# Movement Control Impairment and Low Back Pain: State of the Art of Diagnostic Framing

**DOI:** 10.3390/medicina55090548

**Published:** 2019-08-29

**Authors:** Soleika Salvioli, Andrea Pozzi, Marco Testa

**Affiliations:** Department of Neuroscience, Rehabilitation, Ophthalmology, Genetics, Maternal and Child Health, University of Genova, Campus of Savona, 17100 Savona, Italy

**Keywords:** low back pain, motor control impairment, movement control disease, movement test, reliability, validity

## Abstract

*Background and objectives:* Low back pain is one of the most common health problems. In 85% of cases, it is not possible to identify a specific cause, and it is therefore called Non-Specific Low Back Pain (NSLBP). Among the various attempted classifications, the subgroup of patients with impairment of motor control of the lower back (MCI) is between the most studied. The objective of this systematic review is to summarize the results from trials about validity and reliability of clinical tests aimed to identify MCI in the NSLBP population. *Materials and Methods:* The MEDLINE, Cochrane Library, and MedNar databases have been searched until May 2018. The criteria for inclusion were clinical trials about evaluation methods that are affordable and applicable in a usual clinical setting and conducted on populations aged > 18 years. A single author summarized data in synoptic tables relating to the clinical property; a second reviewer intervened in case of doubts about the relevance of the studies. *Results:* 13 primary studies met the inclusion criteria: 10 investigated inter-rater reliability, 4 investigated intra-rater reliability, and 6 investigated validity for a total of 23 tests (including one cluster of tests). Inter-rater reliability is widely studied, and there are tests with good, consistent, and substantial values (waiter’s bow, prone hip extension, sitting knee extension, and one leg stance). Intra-rater reliability has been less investigated, and no test have been studied for more than one author. The results of the few studies about validity aim to discriminate only the presence or absence of LBP in the samples. *Conclusions:* At the state of the art, results related to reliability support the clinical use of the identified tests. No conclusions can be drawn about validity.

## 1. Introduction

Low Back Pain (LBP) is one of the most frequent health problems causing absenteeism and disability, and it is the most expensive diagnosis in the Western World [1,2,3]. LBP is defined as pain “strong enough to limit normal activities for more than one day” [4] in the lower part of the column, between the 12th thoracic vertebra and the 1st sacral, with possible projection to the lower limb [5].

Temporal staging defines LBP acute when an episode occurred not more than 6 weeks previously, subacute between 6 and 12 weeks, and chronic beyond 3 months [6].

Smoking and obesity have shown a significant association for developing LBP [7], while sedentary lifestyle, low aerobic capacity [8], and psychological factors related to personal or professional discomfort [9] have been indicated as highly related.

Patients with LBP generally improve in the first 6 weeks after an acute episode [10], but approximately 70% of patients show a recurrence in the following year [11,12] while 40% develop chronic LBP [13].

Only in 10–15% of patients with LBP is it possible to identify the triggering factor (root compressions, vertebral fractures, tumors, infections, inflammatory diseases, spondylolisthesis and vertebral stenosis, or proclaimed instability [14]); in the remaining 85–90%, it is difficult to recognize the source of pain. In these cases, the term Non-specific Low Back Pain (NSLBP) is generally used [15,16,17].

Since there is no clear detrimental mechanism identifiable as the source of the disorder, in the last years, researchers focused on identification of subcategories with the aim of developing targeted interventions. The heterogeneity of the samples of study seems to be the basis of the disappointing results obtained in clinical trials that have investigated the management of LBP in the past [18].

The subgroup of patients with motor control impairment (MCI) was proposed for the first time by O’Sullivan [19]. In the literature, different synonyms are used, including movement control dysfunction, movement system impairment or clinical instability, and segmental instability [20].

Patients with MCI tend to experience pain during motor tasks that load the spine mainly in one plane of space. They performed it with unconscious compensation strategies or with the adoption of postures traceable to typical patterns. In order to allow for a more detailed classification and targeted treatment, patients are categorized according to the type of posture and the direction of provocative movement (e.g., flexion pattern and extension pattern) [21].

Strategies for conducting the objective examination are based mainly on the interpretation of the quality of execution of specific tasks or on the use of technology through motion analysis tools. Several tests have been proposed to diagnose MCI, but diagnostic properties have not been thoroughly and conclusively investigated. In order for a classification system to be useful, examiners must be able to determine a valid and reliable individual’s classification.

Reliability is the degree of agreement between a series of measurements of the same occurrence when the measurements are made by changing one or more conditions; validity is the ability of a test to actually measure what the author intended to measure [22].

To date, only two systematic reviews are available, limited purely to the reliability parameters [23,24].

The aim of this review is to summarize the results derived from diagnostic accuracy studies and to update the knowledge about reproducibility in order to provide an exhaustive overview of the state of the art of the diagnostic procedures useful to identify MCI.

## 2. Materials and Methods

No protocol has been previous registered. In order to ensure transparency and reproducibility of the research results, the indications from the PRISMA statement [25] and the COSMIN checklist have been integrated [26].

### 2.1. Eligibility Criteria

#### 2.1.1. Study Design

Primary studies investigating the clinical properties of tests developed to detect MCI have been included. The study design did not influence the decision to include it in this review. Only papers published in English or Italian were considered, with no filters on the date of publication.

#### 2.1.2. Participants Characteristics

The studied population is defined by the presence of NSLBP (with or without lower limb pain) and age > 18 years, without gender distinction.

#### 2.1.3. Test

Only evaluation methods that are easy to use in clinical practice have been considered, excluding examinations that require complex and expensive technological instrumentation.

#### 2.1.4. Diagnostic Values

Properties of tests taken into consideration in the synthesis are validity and reliability, described through the typical coefficients of biomedical statistics.

#### 2.1.5. Data Sources and Search

A systematic search was conducted on the Medline, Cochrane Library, and MedNar (grey or unpublished literature) databases without time filters. The selection of articles can be considered updated to 13 May 2018. Table 1 summarizes the strategy used.

### 2.2. Data Synthesis and Analysis

#### 2.2.1. Study Selection

**Figure 1:**
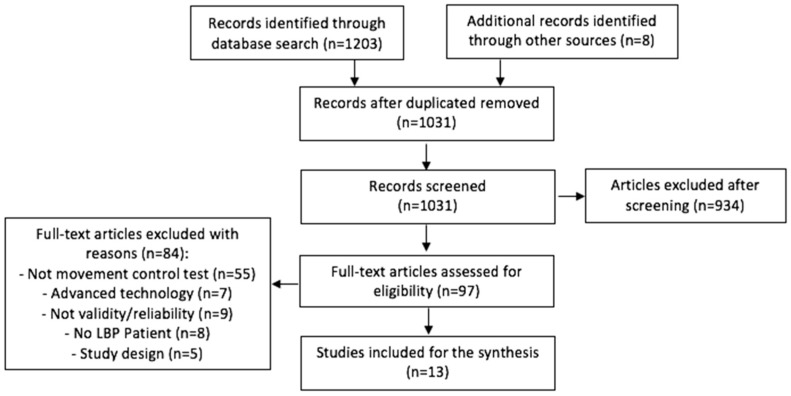
Study selection process.

The studies obtained were initially reported in a comprehensive database, and double reports were excluded. Only one reviewer performed the first screening following the reading of the title and abstracts. Relevance was then assessed by reading the full text: any doubts were resolved with the intervention of a second reviewer. The inclusion process is summarized graphically in a flowchart in the results section (Figure 1). Hand searching has been conducted checking bibliographies of included articles.

#### 2.2.2. Data Extraction and Synthesis

The relevant data were organized in a synoptic tables (Table A1, Table A2, Table A3 and Table A4) which shows author and year of publication, objectives of the study, the characteristics of the participants (number, sex, age, and condition), the characteristics of the examiners, the diagnostic test/examination and the procedure followed, the statistical values, and the main results. No meta-analysis of the collected data was performed, but a narrative synthesis in accordance with the emerging evidence was performed.

#### 2.2.3. Risk of Bias Assessment

The quality of each study was assessed for methodological rigor and risk of bias by one reviewer using the tool described by Brink and Louw [27] (Table A5) and developed for the analysis of validity and reliability studies. Doubtful opinions have been resolved with the help of a second reviewer. This appraisal tool does not incorporate a quality score, but instead, the impact of each item on the study design should be considered individually. This tool contains 13 items, which should be considered according to the nature of the study: 4 are useful only for the evaluation of reliability studies, 4 are useful only for validity studies, and 9 are useful for both. The results were summarized in a synoptic table (Table A1), and a critical discussion of the strengths and weaknesses of the studies included was drafted.

## 3. Results

The database research identified 1203 articles, while 8 others have been identified with free research in the bibliographies of relevant studies for a total of 1211 articles; 180 articles were deleted because they were duplicated, resulting in 1031 basic articles as a partial result. Following the reading of the title, 386 articles were discarded; following the reading of abstract, 548 remained. Following the reading of the full text, 13 studies were included in the review and 84 studies were excluded as not relevant. The steps related to the selection of articles are outlined in the flow-diagram below (Figure 1). Of the 13 studies included, 10 investigated inter-rater reliability [28,29,30,31,32,33,34,35,36,37], 4 investigated intra-rater reliability [31,32,33,38], and only 6 studies analyzed validity [28,29,31,32,39,40]. Overall, the tests showed reliability ranging from fair to excellent (*K* value between 0.32 and 1.00) for the inter-rater and from moderate to excellent for the intra-rater (*K* value from 0.42 to 1.00). The ICC also varied from 0.41 to 0.98, indicating a range from poor to very good (Table A1). A meta-analysis of collected data was not conducted due to the small number of studies that have investigated the same test. In addition to this, the highly heterogeneous nature of the descriptions and the small samples make the calculation superfluous.

### 3.1. Inter-Rater Reliability

The results for inter-observer reliability are shown in Table A2.

Seventeen tests were investigated by a single author [28,30,31,32,33,36,37]. In the remaining 6 evaluated by multiple studies, only 4 (waiter’s bow, one leg stance, sitting knee extension, and prone hip extension) showed agreement between reliability values [29,33,34,36], while for the other 2 (bent knee fall out and active straight leg raising), this did not happen [29,30,33,35,36,37].

#### 3.1.1. Tests Described by More Than One Study that Did Not Give Consistent Results

Bent knee fall out was studied by 3 authors out of 132 patients. It was identified as having modest reliability by Luomajoki et al. [33] (*K* = 0.38) and as poor-excellent by Roussel et al. [36] (ICC = 0.61–0.91) and Enoch et al. [30] (ICC = 0.94). Active straight leg raising has been described in 3 studies on 158 total subjects. Roussel et al. [35] and Bruno et al. [29] showed good reliability (*K* from 0.70 left leg to 0.71 right leg for the first study and 0.79 for the second). Also, Roussel et al. in the study of 2009 [36] provide more variable values, with an ICC from poor to excellent (ICC = 0.41–0.91).

Bent knee fall out was studied by 3 authors out of 132 patients. It was identified as having modest reliability by Luomajoki et al. [33] (*K* = 0.38) and as poor-excellent by Roussel et al. [36] (ICC = 0.61–0.91) and Enoch et al. [30] (ICC = 0.94). Active straight leg raising has been described in 3 studies on 158 total subjects. Roussel et al. [35] and Bruno et al. [29] showed good reliability (*K* from 0.70 left leg to 0.71 right leg for the first study and 0.79 for the second). Also, Roussel et al. in the study of 2009 [36] provide more variable values, with an ICC from poor to excellent (ICC = 0.41–0.91).

#### 3.1.2. Tests Described by More Than One Study that Showed Agreement between the Results

Substantial reproducibility was found for both waiter’s bow (investigated in 2 studies [33,36], 92 subjects, *K* = 0.62 and 0.78) and prone hip extension (investigated in 2 studies [29,34], with 112 total subjects, *K* = 0.72–0.76). The Sitting knee extension was analyzed in 2 studies for a total of 80 subjects. It provided a good *K* in the study by Luomajoki et al. [33] (*K* = 0.72) and was excellent in the study by Enoch et al. [30] (ICC = 0.95). The one leg stance was described in 3 studies for a total of 95 participants. Only Luomajoki et al. [33] identified a moderate-good reliability (*K* = 0.43–0.65), while both Roussel et al. [35] and Tidstrand and Horneij [37] obtained good-excellent values (*K* from 0.75 to 1.00).

#### 3.1.3. Tests Described by a Single Study

Excellent reliability has been identified for joint position sense [30], sitting forward lean [30], and leg lowering [30]. Substantial reliability was identified for pelvic tilt [33], rocking pelvis forwards [33], standing back extension test [31], static lunge test [32], and dynamic lunge test [32]. Moderate reliability was identified for knee lift abdominal test [36], rocking pelvis backwards [33], prone active knee flexion [33], and standing knee-lift test [32]. The unilateral pelvic lift showed moderate reliability for the left side (*K* = 0.47) and substantial for the right side (*K* = 0.61) [37]. The sitting-on-a-ball test, on the other hand, was substantial for the right (*K* = 0.79) but excellent for the left (*K* = 0.88) [37]. The trunk forward bending and return to upright test, described by Biely et al. [28], showed *K* values from 0.35 to 0.89, depending on the criterion used to define the positivity of the test. Also, static lunge test [32], dynamic lunge test [32], and standing knee-lift test [32] showed different reliability values depending on each component observed during the execution of the test.

### 3.2. Intra-Rater Reliability

A total of 13 tests were investigated for intra-examiner reliability (Table A3), all by a single author. Waiter’s bow, pelvic tilt, one leg stance, sitting knee extension, rocking backwards, rocking forwards, prone active knee flexion, and crook lying hip abduction were investigated by Luomajoki et al. [33] on 40 subjects; the standing back extension test was investigated by Gondhalekar et al. [31] on 50 subjects; and the knee-lift abdominal test was investigated by Ohe et al. [38] on 60 subjects. The *K* value is between 0.51 and 0.95, indicating moderate to excellent reliability; it is the same for the good ICC value for Knee lift abdominal test (KLAT) (0.71–0.79). Standing knee-lift test, static lunge test, and dynamic lunge test were studied by Granström et al. [32] and showed good to poor reliability (ICC from 0.54 to 0.87). In the same study, the intra-examiner reliability of different aberrant movements analyzed during the execution of the above 3 tests was also investigated, and in this case, an extreme variability in the results also emerged (*K* from 0.42 to 1.00).

### 3.3. Validity

A total of 10 tests (including batteries) have been reported with indicating their validity, represented in Table A4 and all investigated by a single author. The battery of Luomajoki et al. [39], the knee-lift abdominal test, the bent knee fall out, the prone hip extension, and the active straight leg raise showed significant relationships between test positivity and the presence of LBP compared to healthy subjects (all *p* < 0.05). The use of Judder/shake/instability catch (JUD), deviation from sagittal plane (DEV)and aberrant movement score (AMS) as positive criteria in anterior trunk flexion movement and return to upright position also showed significant correlations with the presence of LBP. On the contrary, for the standing back extension test, standing knee-lift test, static lunge test, and dynamic Lunge, test there were not enough high values of diagnostic power (AUC from 0.47 to 0.78).

### 3.4. Risk of Bias in Included Studies

All studies included reported a complete description of the selected sample (Table 2). In several [29,34,37,38], however, there was no method for calculating the sample size, so we do not know with certainty the statistical power of the results obtained. The presence of an adequate method for calculating the sample size was not described as a parameter to be evaluated in criterion 1, and for this reason, it was considered satisfied in all the studies. Three studies [36,38,40] did not clarify the characteristics of the evaluators. The main source of risk of bias in 8 out of 11 studies dealing with reproducibility was the simultaneous evaluation by the observers [34,37]. Three studies did not clarify or carry out the randomization of the order of the patients evaluated [33,36,39]. Four did not randomized the order of the tests administered [30,33,37,39], and one did not clarify it [36]. In addition, in 2 studies, the blindness of the evaluators to the results between them was not clearly explained [36,37]. In studies dealing with intra-operator reproducibility, the concealment of patients or an adequate time gap between the two observations was adopted, except for 1 study [38], where the assessments were re-performed in a matter of minutes. In the studies that dealt with validity, they were not met or it was not possible to judge the criteria (3,7,9,11) because there is no shared reference in the literature. Analyses of diagnostic accuracy were developed with respect to the presence of LBP or not. Only 1 study [31] gave a description of the reference standard used, but in our opinion, the choice was not appropriate. The choice of statistical methods was considered appropriate for all studies; only 1 study [34] introduced a possible distortion of the effect of the results because it presented data of a nonparametric nature by inserting the standard deviation.

## 4. Discussion

This review is the first to include and summarize results from reliability (inter- and intra-rater) and validity studies of tests designed to detect MCI in subjects with NSLBP.

In 2013, Carlsson and Rasmussen-Barr [23] studied the reliability of tests to diagnose MCI and found it difficult to identify consistent results because they were investigated by studies with a high risk of bias. At the time (with a research updated to October 2011) only prone knee bend and the one leg stance were indicated by the author as useful because they were presented in one study with a low risk of bias. Recently, Denteneer et al. [24] identified a greater number of tests (specifically 30) but the limit of his research were the inclusion criteria. Studies included populations classified with functional lumbar instability or MCI or with the association of both. This leads to sampling limits with difficult interpretation and comparison of results.

In the present research, 15 tests have shown good inter-examiner reliability in at least one study, but only waiter’s bow, one leg stance, sitting knee extension, and prone hip extension had almost overlapping values in at least 2 studies.

As is well known, inter-rater reliability is just a component of the reliability of a test and take greater importance when its context of use is characterized by the alternation of operators. NSLBP rehabilitation process is in most cases managed by a single therapist; nevertheless, the number of studies that have dealt with intra-rater reliability is far less than those of the inter-rater reliability.

From the few data available, there seems to be a good degree of agreement in the case of repeated measurements by the same therapist for almost all tests. The summary of results about intra- and inter-rater reliability shows that observing abnormal movement strategies in patients with NSLBP seems to be possible through simple tests; anyway, positivity criteria and execution modalities need to be standardized with precise protocols, as suggested by Enoch et al. [30].

The clinical use of the tests has to be based on consistent evidence both for the intra/inter-rater reliability, and these conclusions must derive from at least 2 studies of good quality.

Compared to knowledge set by Carlsson and Rasmussen-Barr [23], we can still recommend the use of the one leg stance, but we add also waiter’s bow and sitting knee extension for the low risk of bias of the studies. These 3 tests are the only ones to have been studied both for inter-rater and intra-rater reliability. The use of prone knee bend suggested by Carlsson and Rasmussen-Barr [23] is less corroborated because, to date, it remains investigated only by one author and values of inter-rater reliability are moderate. Prone hip extension cannot be recommended due to high risk of bias in one of the two studies in which it is investigated. Moreover, there are no studies available about intra-rater reliability for prone hip extension.

Since 2011, the literature did not add much to previous knowledge, because of both the number of studies published and the quality of them.

As well as for the intra-rater reliability, the studies that have dealt with the validity are few in number. There is not a single test that has been evaluated by more than one author. The studies included in this review show that most tests are able to distinguish only subjects with LBP from healthy subjects (knee-lift abdominal test, bent knee fall out, and trunk forward bending and return to upright) [28,40]. This means that they do not provide any additional information to that which may result from a well-conducted medical history. It must be said that, in general, there is a higher sensitivity of the tests [39] towards subjects with chronic LBP, suggesting an association between the duration of symptoms and MCI, which would require observational studies to be demonstrated. At the same time, more patients with a history of LBP than healthy subjects [28] were positive, indicating the possibility that MCI may persist over time despite the resolution of symptoms. Again, only the design of ad hoc cohort studies could demonstrate the relationship between MCI and recurrence due to possible overloading of the tissues of the lower spine.

The validity data also shows the small number of researches that dealt with the diagnostic procedures aimed at identifying directional patterns of MCI [31]. The most important barrier to the development of validity research is the absence of a golden standard to compare the same outcome with different methods of investigation. Considering that tests for MCI evaluate the performance of certain motor tasks, the use and validation of motion capture tools seems to be the most appropriate strategy to make the evaluation as objective as possible. To date, only Wattananon et al. [41] has tried to establish reference values for the interpretation of clinical trials through comparison between the observation of examiners and the digital data collected.

Summarizing, only waiter’s bow, sitting knee extension, and one leg stance are assessed across studies of good quality with good-excellent values both for intra-rater and inter-rater reliability; therefore, their use in clinical practice may be considered. However, the main problem remains the lack of clarity about the validity, which today, does not allow conclusions on the accuracy of the subgrouping procedure.

## 5. Conclusions

Implications for clinical practice:

- Inter-rater reliability is widely studied. Waiter’s bow, prone hip extension, sitting knee extension, and one leg stance showed good values confirmed by at least two studies;
- Intra-rater reliability is not largely investigated. From the few studies available, good repeatability values seem to emerge;
- Only waiter’s bow, sitting knee extension, and one leg stance are assessed across studies of good quality with good-excellent values both for intra-rater and inter-rater reliability;
- There is a lack of evidence regarding the validity of MCI tests, which results from diagnostic accuracy analyses aimed at discriminating only the presence or absence of LBP in the study samples;
- Final conclusions regarding the clinical and scientific use of the identified tests can be drawn only when consistent values of reliability and validity can be found in the literature.

Review limitations:

- Processes of identification, selection, evaluation, and data collection were carried out by a single author, contrary to the indications contained in the PRISMA statement. Intervention of a second author was required only in case of doubt;
- Inclusion of studies published only in Italian and English;
- Absence of protocol registration procedure.

Review strengths:

- Inclusion of grey literature.

Implications for research and future research:

- Investigate further intra-rater reliability of MCI tests in patients with NSLBP;
- Indicate subgroups of patients with NSLBP having salient characteristics related to MCI and deductibles in history. Develop an analysis of diagnostic accuracy of tests for motor control as a function of them;
- Identify a gold standard to evaluate the diagnostic accuracy of individual tests;
- Standardize protocols for the preparation, execution, and evaluation of the tests in order to allow a comparison between them and the generalization of the results.

## Figures and Tables

**Table 1:** Search strategy used for every database.

Database	Search Strategy
MEDLINE—Clinical queries	Low Back Pain AND motor control
	(Impairment AND (motor control OR movement OR movement control OR movement coordination OR movement system OR muscle control OR trunk motor control)) OR (Dysfunction AND (movement control OR movement OR stability)) OR (deficit AND (movement precision OR trunk muscle timing OR trunk movement control)) OR MCI OR altered sensory function OR segmental instability) AND (Low Back Pain OR LBP OR non-specific low back pain OR NSLBP)
Cochrane Library—Simple Search	Low Back Pain AND motor control
MedNar—Simple Search	Low back pain AND motor control

**Table 2:** Risk of bias summary.

Question and Nature of the Study	[34]	[33]	[35]	[39]	[36]	[37]	[30]	[40]	[28]	[29]	[38]	[31]	[32]
1. Human subjects and detailed description of the sample (validity and reliability studies)	Y	Y	Y	Y	Y	Y	Y	Y	Y	Y	Y	Y	Y
2. Qualification or competence of rater/s clarified (validity and reliability studies)	Y	Y	Y	Y	N	Y	Y	N	Y	Y	N	Y	Y
3. Reference standard explained (validity studies)	N/A	N/A	N/A	N	N/A	N/A	N/A	N	N	N	N/A	Y	N
4. Blinding of raters to the findings of other raters (inter-rater reliability studies)	Y	Y	Y	N/A	N	N	Y	N/A	Y	Y	N/A	Y	Y
5. Blinding of raters to their own prior findings (intra-rater reliability studies)	N/A	Y	N/A	N/A	N/A	N/A	N/A	N/A	N/A	N/A	N	Y	Y
6. Variation in order of examination (reliability studies)	N	N	Y	N	N	N	Y	N/A	N	N	N	Y	Y
7. Latency between application of reference and index test reasonably (validity studies)	N/A	N/A	N/A	N/A	N/A	N/A	N/A	N	N	N	N/A	Y	N
8. Stability of the variable considered before repeated measures (reliability studies)	Y	Y	Y	N/A	Y	Y	Y	N/A	Y	Y		Y	Y
9.Reference standard independent of the index test (validity studies)	N/A	N/A	N/A	N	N/A	N/A	N/A	N	N	N	N/A	Y	N
10. Detailed description of index test (validity and reliability studies)	Y	Y	Y	Y	Y	Y	Y	Y	Y	Y	Y	Y	Y
11. Detailed execution of reference standard (validity studies)	N/A	N/A	N/A	N	N/A	N/A	N/A	N	N	N	N/A	Y	N
12. Explanation of the withdrawals (validity and reliability studies)	Y	Y	Y	Y	Y	Y	Y	N	Y	Y	N	Y	Y
13. Appropriateness of statistical methods (validity and reliability studies)	N	Y	Y	Y	Y	Y	Y	Y	Y	Y	Y	Y	Y

## Appendix A

**Table A1:** Characteristics of included studies.

Study	Aim	Population Characteristics	Examiners Characteristics	Methods	Outcomes	Results
Murphy et al. [34]	To investigate whether the finding of deviation of the lumbar spine during the hip extension test could be detected reliably by clinicians trained in the performance of the test	*N* = 42 (31 W) with LBP > 7 weeks	Two chiropractic physicians: (1 with 13 years of experience and 1 with <1 year of experience) and a training period pre-study of 1 h.	Hip extension test for each hip. Max 3 repetitions.	Dichotomous judgment (Test +/−)	*K* = 0.72 (L)–0.76 (R)
		Average age 37.8 (range 19–60).		Observers evaluate the patient at the same time and are “blind” to the results of the colleague’s evaluation.	*K* coefficient for inter-operator reliability	
		Patients from spinal center.				
Luomajoki et al. [33]	To determine the inter- and intra-operator reliability of 10 MCI tests of the lumbar spine.	*N* = 40 (26 D, 14 U).	4 examiners with 3-day of intensive course on MCI prior to assessment.	10 MCI tests:	Dichotomous judgment (Test +/-); *K* coefficient for inter-and intra-operator reliability	Inter-rater:
		13 LBP + 27 healthy.		Waiter’s bow, pelvic tilt, one leg stance R, one leg stance L, sitting knee extension, rocking backwards, rocking forwards, dorsal tilt of pelvis, prone active knee-flexion, and crook lying.		
						*K* = 0.38–0.72
		Average age: 52.1.	2 examiners were specialists in MCI and had postgraduate degrees in manual therapy, with 25 years of working experience. The other	Raters were blinded to the diagnosis of patients and the colleagues’ evaluation results. The performances were recorded (anonymously), and raters watched each video only once.		
						Intra-rater:
		Patients from private physiotherapy practice.				
						*K* = 0.51–0.95
			2 raters were Pt with 5 years of experience.	Reviewed after 2 weeks.		
Roussel et al. [35]	To investigate reliability and internal consistency of 2 clinical tests that analyze motor control mechanisms.	*N* = 36 (21 W) with LBP	2 examiners: 1 with master’s degree and 1 Pt with 4 years of clinical experience.	Trendelenburg	Dichotomous judgment (Test +/−)- weighted *K* for inter-operator reliability	*K* = 0.70–0.83 for Trendelenburg and ASLR.
				Active straight leg raise		
		Average age (mean ± SD): 37.4 ± 11.6 (range 21–62)	Training of 2 h x 2 days by an expert + evaluation of 10 pre-study patients.	Evaluation by examiner 1, 10’ rest (in which the patient was asked to complete questionnaires), then evaluation by examiner 2.		
		Patients from a private clinic and 2 outpatient physiotherapy clinics.				
				Order of the tests randomly assigned.		
				Both examiners were blinded to the others’ scores and the patients’ medical history.		
Luomajoki et al. [39]	To evaluate the performance of 6 MCI tests in LBP and healthy patients.	*N* = 210 (130 W, 80 M)	12 examiners with 7 years of average working experience, all with OMT specialization.	Cluster of 6 tests:	Dichotomous judgment (Test +/−) N° of test +	N° of positive tests: 2.21 in LBP group and 0.75 in healthy controls.
				Waiter’s bow, pelvic tilt, one leg stance, sitting knee extension, rocking 4 point kneeling, and prone knee bend.		
	Understand whether staging of LBP affects the results.	102 healthy, 108 LBP:	Raters were trained using instruction, patient cases, and rating of videotaped tests.	The order of the tests was always the same.	Effect size for the difference between group	Effect size between-group: 1.18 (95% CI: 1.02–1.34), *p* < 0.001.
		29 with LBP <6 weeks, 30 with 6–12 weeks, 46 with LBP >12 weeks.				
		Patients from 5 physiotherapy clinics.		Pt were not blinded to the patient’s group.		
Roussel et al. [36]	To determine inter-ex reliability and internal consistency of the 4 clinical tests examining lumbopelvic MCI in patients with and without LBP.	*N* = 52	With three 1-h training sessions, 2 examiners were trained in performing the tests under supervision of 2 manual therapists.	MCI evaluation with PBU: -Active straight leg raising, bent knee fall out, knee lift abdominal test, and standing bow.	ICC	ICC = 0.41–0.91
		25 healthy, 27 with LBP (>3 months).			*K* coefficient	*K* = 0.78 (healthy) e 0.80 (LBP)
				Observation examiner 1 → 10-min rest → observation examiner 2. Assessors were blinded to the medical history of the patients.	Chronbach α for internal consistency	Chronbach α = 0.83 (LBP) e 0.65 (healthy).
Tidstrand and Horneij [37]	To determine inter-examiner reliability of 3 tests of muscular functional coordination of the lumbar spine in patient with LBP.	*N* = 19 (9 W, 10 M)	2 experienced Pts, both trained in orthopedic manual therapy and in the McKenzie method. Both had more than 5 years of experience of treating patients with lumbar instability.	The 2 examiners evaluated individually but simultaneously the patients in the following tests:	Dichotomous judgment (Test +/−) - *K* of Cohen for inter-ex reliability	*K* range = 0.47–1.00
				le-Single limb stance, sitting on Bobath ball with one leg lifted, and unilateral pelvic lift.	% of agreement	
		13 with LBP.	Pre-study trial on 10 patients.	Each test was performed once on both sides, and each test position was maintained for 20 s. Tests were administered in the same order to all patients.		
		Average age ± SD: 42 years ± 12.				mean *K* = 0.77.
		Patients from a private physiotherapy clinic.		Examiners were blinded to the patient’s symptoms.		
				Detected the VAS score before each test: VAS > 7/10 was an exclusion criterion.		
Enoch et al. [30]	To determine inter-operator reliability of MCI tests on patients with and without LBP	*N* = 40 (26 W, 14 M).	2 examiners with 20 years of clinical experience, teachers at the Danish Manual Therapy Society.	Each patient was evaluated by each operator independently in two separate rooms. Both examiners performed the tests in the same order on each subject.	total mean + standard deviation for each test.	ICC = 0.90–0.98
		LBP 25 + 15 healthy.				
		Age range: 20–82.				
		Patients from 3 private clinics of physical therapy.	Pre-study trial on 10 patients.	5 tests for MCI:	ICC for inter-ex reproducibility	Mean ICC = 0.95
				Joint position sense, sitting forward lean, sitting knee extension, bent knee fall out, and leg lowering. Max 10 repetitions of each test.		
Roussel et al. [40]	To compare lumbopelvic motor control between dancers with and without a history of LBP.	*N* = 40 (38 W, 2 M)		2 tests were used for evaluation of MCI:	mmHg pressure on PBU and difference between groups	*p* = 0.048 KLAT
		Age 17–26. Mean age 20.3 (SD 2.4).				
		16 patients with LBP (at least 2 consecutive days in the last year).		Knee lift abdominal test,		*p* = 0.049 BKFO
				Bent knee fall out.		
		Patients from the Department of Dance of a Conservatoire in Belgium.		The tests were performed in supine position and monitored with a PBU.		
Biely et al. [28]	To investigate the inter-examiner reliability of observation of aberrant movement patterns and whether each pattern is associated with current LBP.	*N* = 102 (48–57% D)	5 examiners with experience from 5 to 25 years in orthopaedic examination of the low back, including 2 certified orthopaedic clinical specialists.	2 therapists simultaneously observed the patient perform 3 repetitions of trunk forward bending and return to upright for the presence of the following 3 aberrant movement patterns:	Dichotomous judgment (Test +/−)	*K* = 0.35–1.00
						Construct validity: LBP vs no LBP:
						*p* = 0.004 DEV
						*p* = 0.002 JUD
						LBP vs LBP history: * p* = 0.001 JUD
						No LBP vs history LBP: *p* = 0.001 DEV
						AMS:
						*p* < 0.001 for
						LBP
				Altered lumbo-pelvic rhythm (including Gower’s sign), deviation from the sagittal plane (DEV), instability catch (JUD).		No LBP vs LBP
						LBP vs history LBP
						*p* = 0.021 for No LBP vs history LBP
		Average age: 41.1–44.4				
		35 without LBP, 31 with current LBP, 36 with history of LBP.			*K* value for inter-examiner reliability	
					*p* value as correlation index for construct validity.	
		Patients from 2 physiotherapy clinics.	2 h of pre-study training and a study manual.			
				Examiner blinded to group membership. Each therapist’s observations were recorded on a separate clinical observation of aberrant movement form. No discussion between raters.		
Bruno et al. [29]	To investigate: the difference between LBP subjects and healthy in	*N* = 70 (40 W, 30 M)	2 chiropractors with over 30 years of clinical experience.	The participants performed 3–5 repetitions of each test, while the examiners simultaneously observed the performances:	Dichotomous judgment (Test +/-)- score 0–5 for the participant-reported perception of difficulty	PHE: *K* = 0.72
						ASLR: *K* = 0.79
						Participant scores (average):
	reported perception of difficulty in the test execution and;	Average age 27.7 years old.				PHE:
				Prone hip extension (PHE),		1.33 (0.11) LBP
						0.38 (0.07) healthy.
				Active straight leg raise (ASLR).		ASLR:
	participant difference in reported perception of difficulty between subjects rated as positive or negative.	30 with LBP, 40 healthy.				
						0.85 (0.11) LBP
						0.25 (0.05) healthy.
					*K* for inter-ex reliability	PHE and ASLR:
				The order of the test and leg lifted first were randomized.	Sensitivity and specificity	*p* < 0.001 for group status and participant scores. Not between group and examiner classification. Not between examiner classification and participant scores.
						LBP group perceived significant difficulty compared to the control group.
						PHE:
	- specificity and sensitivity of participant-reported perception of difficulty scores in individuals with non-pregnancy-related LBP and controls.	Patients from local medical, chiropractic, physiotherapy, and massage therapy clinics	Pre-study: 1 meeting and 3 training session to achieve a consensus.	The examiners were blinded to the group status and to the colleague’s score.		Sn: 0.82–Sp: 0.69
						ASLR:
				Patient were blinded to the evaluation of the examiners, and they were asked to express a score on a scale of 0–5 after the observer had left the room.		Sn: 0.60–Sp: 0.76. (in cut-off 0–1).
Ohe et al. [35]	To quantify the characteristics of the trunk control during active limb movement in LBP patients with different types of LBP manifestation based on direct mechanical stress to the lumbar spine.	*N* = 60 (33 W, 27 M).	1 examiner which instructs the patient to perform the test.	During the unilateral leg-raising movement in crook-lying position (for 3 times), pressure changes produced by the movement of the lumbar lordotic curve were measured by a PBU.	ICC were calculated to confirm the relative reliability	ICC = 0.71–0.79
		Age 20–58				
		30 LBP, 30 healthy.		Data collection was executed 4 times. These 4 trials provided 4 repetitive sets of data of back pressure. Each trial was performed with 30 s rest.		
		Patients from the outpatient department of the local hospital.				
Gondhalekar et al. [31]	To determine the intra- and inter-rater reliability and concurrent validity of the standing back extension test for detecting MCI of the lumbar spine.	*N* = 50.	2 examiners with OMT specialization.	All patients were assessed in two observations that were 24 to 48 h apart at the same time of day by both operators separately. Both the raters took two readings for each subject in two different visits.	Dichotomous judgment (Test +/-)	Intra-rater:
						*K* = 0.87
						% agreement: 96
					For reliability:	Inter-es:
						*K* = 0.78
					% agreement	% agreement: 94
		25 with NS-LBP, 25 healthy controls.		Finally, they underwent evaluation by ultrasound as a gold standard.	*K* coefficient.	AUC 0.785 for ADIM 0.780 for ASLRs
				Order of examination was varied.	For validity:	
				Both raters were blinded to the findings of the other rater and to their own prior findings.	Test +/-	
		Age 32.6–33.5			Area under the curve (AUC)	
				Raters were not blinded to the subject’s disease status.	Sn and Sp	
					LR	
Granström et al. [32]	To evaluate inter- and intra-examiner reliability and discriminative validity of 3 movement control tests.	*N* = 38 (24 W, 14 M).	4 examiners with 13–32 years’ work experience, all were qualified orthopedic manual therapists.	Patients performed 3 tests in a standardized order:	For inter and intra-ex reliability: ICC	Inter-observer: ICC = 0.68–0.80.
						Intra-observer: ICC = 0.54–0.82
				Standing knee lift (SKL), static lunge (SL), and dynamic lunge (DL).		
				They were video recorded on the frontal and sagittal planes.	For validity: ROC curves	Validity ranged between 0.47 and 0.56.
				The examiners (blinded to the subjects’ health status and each other’s results) individually scored the tests and calculated a composite score for each test based on the number of incorrect test components (0 or 1).		
				For inter-observer reliability, the observers received the numbered video clips (a random-drawn number showing which of the video clips to begin with).	AUC	SKL not-informative, SL and DL are less accurate than the effect of chance alone in discriminating subjects into healthy or NS-LBP group.
		Average age 37.5 years (19–58). 21 NSLBP, 17 healthy.	Pre-study: one-day course in evaluating the tests + training session and test trial on video clips.	They were instructed to study each video clip no more than five times. The same procedure was repeated after 2 weeks.		
		Patients with LBP from private physiotherapy clinics, the healthy selected from university students and acquaintances.				

**Table A2:** Inter-rater reliability of clinical tests.

Test	Authors	Reproducibility INTER-ES	Percentage of Agreement	Description	Positivity Criteria
Active Straight Leg Raising (ASLR)	Bruno et al. [29] ***	*K* = L: 0.70		In supine position, hip flexion with fully extended knee required.	* Expressed the perceived difficulty on a scale of 0–5
		R: 0.71			
		ICC = 0.41–0.91			** Observation of the difference in mmHg from the starting phase, through the PBU positioned behind the column.
	Roussel et al. [35] *	Cronbach α = 0.83			
					*** The examiner determines the positivity/negative of the test according to the subject’s ability to maintain neutral alignment.
	Roussel et al. [36] **	*K* = 0.79			
Crook lying hip abduction/bent knee fall out (BKFO)	Luomajoki et al. [33] *	*K* = 0.38	P1 = 78.6	Supine with hip and knee flexed, required abduction/extra rotation of hip	* Execution evaluated as qualitatively correct by the examiner after careful observation.
					** A pressure biofeedback (PBU) was placed behind the column and evaluated the pressure variation.
	Enoch et al. [30] ***	ICC = tra 0.61 e 0.91	P2 = 65.0		
					*** A 5-cm tape is placed between the two antero-superior iliac spine, with a laser pointer on the right end of the line. After 5 movements, the distance between the laser pointer and the extremity 0 of the tape (in cm) is measured.
		Cronbach α = 0.83			
	Roussel et al. [36] **	ICC = 0.94	88		
Dynamic lunge test (DL)	Granström et al. [32]	ICC = 0.80 (0.68–0.89)		In an upright position, required the functional movement of front lunge and evaluated the dynamic execution with upper limbs in full elevation.	Appearance of compensation. Assess each of the 6 components of the test as correct (1 point) or incorrect (0). A final score is obtained by combining the individual components.
		*K* = 0.45 (0.16–0.73) for trunk lateral flexion (TLF)			
				TLF: Trunk lateral flexion to either side.	
				KMI: The front knee moves inwards and not aligned with the hip and foot PT: The pelvis tilts to either side and not horizontally aligned.	
		*K* = 0.50 (0.22–0.78) for knee moving inwards (KMI)			
				HMB: The hips move backwards instead of downwards. The back seems to arch.	
		*K* = 0.54 (0.28–0.81) for pelvic tilt (PT)		TMF: The trunk moves forwards and falls over the front leg.	
				SMB: The shoulders move backwards when returning back to start position.	
		*K* = 0.46 (0.18–0.75) for hips moving backwards (HMB)			
		*K* = 0.55 (0.29–0.82) for trunk moving forwards (TMF)			
		*K* = 0.77 (0.57–0.97) for shoulders moving backwards (SMB)			
Knee lift abdominal test (KLAT)	Roussel et al. [36]	ICC > 0.85		In supine position, with flexion of knees and hips, flexion of a hip is required.	Difference in the pressure variation between the performance carried out with the two lower limbs
		Cronbach α = 0.83			
Leg lowering (LL)	Enoch et al. [30]	ICC = 0.98		Required to maintain constant pressure on the PBU during repeated lowering of the leg towards the support surface, starting with hips flexed at 90 degrees and knee extended as much as possible.	Difference in the pressure variation between the performance carried out with the two lower limbs.
One leg stance/Trendelenburg	Luomajoki et al. [33] *	*K* = R: 0.43	P1 = R/L: 88.0	One leg balance required	* Lateral displacement of the asymmetrical navel and difference of >2 cm between the two sides
		L: 0.65			
	Roussel et al. [35] **	*K* = R: 0.75	P2 = R: 97.5		
		L: 0.83	L: 92.5		**Appearance of pelvic tilt or rotation or inability to maintain position for 30 s
	Tidstrand and Horneij [37] **	*K* = R: 1.00	R: 100		
		L: 0.88	L: 95		
Pelvic tilt	Luomajoki et al. [33]	*K* = 0.65	P1 = 80.0	Request for anti and retroversion of pelvis	Presence of compensatory movements in others anatomical districts or inability to do the task required
			P2 = 92.5		
Prone active knee flexion/prone knee bending	Luomajoki et al. [33]	*K*(Est) = 0.47	P1 = (Est) 97.6	Keeping the lumbar spine in neutral position lying prone, knee flexion required	Loss of neutral position before 90° knee flexion
			(Rot) 90.5		
		*K*(Rot) = 0.58	P2 = (Est/Rot) 87.5		
Prone hip extension (PHE)	Bruno et al. [29] *	*K* = L: 0.72		Patient in prone position, hip extension with fully extended knee required	* Appearance of rotation, hyperextension, or inclination of the lower spine or pelvic tract. Considered also the difficulty perceived during the execution indicating a score from 0 to 5 (where 0 indicates no difficulty and 5 impossibility to perform) in the overall assessment
		R: 0.76			
	Murphy et al. [34]	*K* = 0.72			
Repositioning (RPS)/joint position sense	Enoch et al. [30]	ICC = 0.90		In an upright position, the patient is asked to search for the neutral lumbar position, following a maximum antiversion and retroversion of the pelvis.	A 5-cm tape positioned vertically starting from S1 (point 0) on which a laser is pointed. The patient moves the pelvis twice in anti and retroversion, finally returning to the starting position. The distance in cm between the laser pointer and S1 is measured.
Rocking backwards	Luomajoki et al. [33]	*K* = 0.57	P1 = 88.0	Keeping the lumbar spine in neutral position, knees and hips flexion required starting from quadrupedic position	Loss of neutral position or appearance of compensation
			P2 = 90.0		
Rocking forwards	Luomajoki et al. [33]	*K* = 0.68	P1 = 92.8	Keeping the lumbar spine in neutral position, knees and hips extension required starting from quadrupedic position	Loss of neutral position or appearance of compensation
			P2 = 92.5		
Sitting forward lean (SFL)	Enoch et al. [30]	ICC = 0.96		Required flexion of the trunk in a seated position, without losing neutral position of the lumbar spine. The distance measured between two points marked on the patient’s skin (point 0 on S1 and point 1 placed 10 cm above).	Increased distance between the two points from the starting position
Sitting knee extension (SKE)	Enoch et al. [30] **	*K* = 0.72	P1 = 90.4	Required to maintain neutral lumbar spine position during knee extension with patient sitting on the edge of the cot	* Capable of maintaining the neutral position of the lumbar spine up to 30–50° knee flexion.
					** A 5-cm tape is placed on the lumbar area starting from S1, on which a laser pointer is placed. After 5 full knee extensions, the distance in cm between the laser pointer and S1 is measured.
	Luomajoki et al. [33]	ICC = 0.95	P2 = 95.0		
Sitting on a ball	Tidstrand and Horneij [37]	*K* = R: 0.79	R: 89	Sitting on a Bobath ball, required to lift one foot off the ground by at least 5 cm.	Occurrence of compensatory movements at the level of the pelvis and trunk or loss of the neutral position of the lumbar spine
		L: 0.88	L: 95		
Standing back extension test	Gondhalekar et al. [31]	*K* = 0.78	94	Request for extension hip with fully extended knee in an upright position	Occurrence of ipsilateral superior anterior iliac spine forward translation or compensatory movements.
Standing knee-lift test (SKL)	Granström et al. [32]	ICC = 0.68 (0.47–0.82)		In an upright position, required flexion of hip and knee at 90°, remaining in monopodal balance, with upper limbs abducted at 90 degrees and elbows extended.	Appearance of compensation. Assess each of the 7 components of the test as correct (1 point) or incorrect (0) A final score is obtained by combining the individual components.
		*K* = 0.32 (0.02–0.63) for hip hitch (HH)		Hip hitch (HH): instead of lifting the thigh up in the sagittal plane, the pelvis tilts in the frontal plane.	
		*K* = 0.67 (0.43-0.90) for lateral sway (LS)		LS is a lateral sway of the pelvis on the stance leg.	
		*K* = 0.77 (0.57–0.97) for trunk lateral flexion (TLF)			
				TLF: Trunk lateral flexion to either side.	
		*K* = 0.48 (0.20–0.76) for knee not lifted straight up (KNLSU)		KNLSU: Knee is not lifted straight up.	
		*K* = 0.83 (0.66–1.00) for arm lowering (AL)		AL: One arm is lower on one side.	
		*K* = 0.91 (0.78–1.00) for back extension (BE)		BE: The back extends during the movement.	
		*K* = 0.68 (0.44–0.91) for back flexion (BF)		BF: The back flexes during the movement.	
Static lunge test (SL)	Granström et al. [32]	ICC = 0.79 (0.65–0.88)		In an upright station, required the functional movement of the front lunge and evaluated the ability to maintain it with upper limbs abducted at 90° and elbows extended.	Appearance of compensation. Assess each of the 5 components of the test as correct (1 point) or incorrect (0) A final score is obtained by combining the individual components.
		*K* = 0.61 (0.35–0.86) for trunk lateral flexion (TLF)		TLF: Trunk lateral flexion to either side.	
		*K* = 0.91 (0.78–1.00) for arm lowering (AL)		AL: One arm is lower on one side.	
		*K* = 0.59 (0.33–0.84) for knee moving inwards (KMI)		KMI: The front knee moves inwards and not aligned with the hip and foot.	
		*K* = 0.67 (0.43–0.90) for pelvic tilt (PT)		PT: The pelvis tilts to either side and not horizontally aligned.	
		*K* = 0.49 (0.21–0.77) for hips backwards (HMB)		HMB: The hips move backwards instead of downwards. The back seems to arch.	
Unilateral pelvic lift	Tidstrand and Horneij [37]	*K* = R: 0.61	R: 79	In supine position, with hips and knees bent, required to lift pelvis from the cot, supporting it on just one foot.	Occurrence of compensatory movements at the level of the pelvis and trunk or loss of the neutral position of the lumbar spine
		L: 0.47	L: 74		
Waiter’s bow/standing bow (SB)	Luomajoki et al. [33] *	*K* = 0.62	P1 = 85.7	Required hip flexion with lumbar spine in neutral position.	Loss of neutral position of the lumbar spine a:
					* 50–70° flexion of the hips.
	Roussel et al. [36] **	*K* = 0.78	P2 = 75.0		** Approx. 50° hip flexion.
Trunk forward bending and return to upright	Biely et al. [28]	For JUD:		During forward bending of the patient and return to upright standing, the examiner observes any aberrant movement pattern:	* Result calculated considering the test as positive if at least 1 movement on 3 repetitions is altered.
		*K* = 0.35 (0.00–0.71) *	96		
		*K* = 0.46 (0.31–0.61) **	96		
		For DEV:			
		*K* = 0.68 (0.34–1.00) *	87		
		*K* = 0.60 (0.50–0.69) **	80	JUD = Judder/shake/instability catch. In an attempt to return from flexion, the patient flexes their knees or moves their pelvis anteriorly before reaching the upright position of the trunk.	
		For altered LPR:		DEV = Deviation from sagittal plane. Considered positive if any deviation from the sagittal plane appears during movement.	
		*K* = 0.89 (0.69–1.00) *	96		
		*K* = 0.83 (0.73–0.93) **	96		
		For battery:		LPR = Reversal of lumbopelvic rhythm (including Gower’s sign). In an attempt to return from flexion, the patient flexes their knees and moves their pelvis anteriorly before reaching the upright position of the trunk.	** Result calculated considering the test as positive only if the movement is altered in each repetition
		*K* = 0.65 (0.00–1.00) *	96		
		*K* = 0.53 (0.43–0.64) **	80	Battery test considered positive for the presence of at least 1 out of 3 of the aberrant movements between JUD, altered LPR and DEV.	

**Table A3:** Intra-rater Reliability of clinical tests.

Test	Authors	INTRA-RATER Reliability	Percentage Agreement/Description
Crook lying hip abduction/lateral rotation	Luomajoki et al. [33]	*K* = 0.86	O1/O2 = 97.5
Dynamic lunge test (DL)	Granström et al. [32]	ICC = 0,54–0,82	The trunk moves forwards (TMF) and falls over the front of the leg.
		*K* = 0.47–0.79 for Trunk Lateral Flexion	
		*K* = 0.63–0.74 for Knee moving inwards	
		*K* = 0.68-0.89 for Pelvic Tilt	
		*K* = 0.47–0.90 for Hips moving backwards	The shoulders move backwards (SMB) when returning back to the start position.
		*K* = 0.64–0.95 for trunk moving forwards	
		*K* = 0.79–0.95 for shoulders moving backwards	
Knee lift abdominal test (KLAT)	Ohe et al. [38]	ICC=0.71–0.79	
One leg stance/Trendelenburg	Luomajoki et al. [33]	*K* = R:0.67 L: 0.84	O1 = R: 92.5
			L: 87.5
			O2 = R/L:100
Pelvic tilt	Luomajoki et al. [33]	*K* = 0.80	O1/O2 = 95.0
Prone active knee flexion/prone knee bending	Luomajoki et al. [33]	*K*(Ext) = 0.70	O1 = (Ext/Rot) 92.5
		*K*(Rot) = 0.78	O2 = (Ext) 92.5—(Rot) 100
Rocking backwards	Luomajoki et al. [33]	*K* = 0.72	O1/O2 = 97.5
Rocking forwards	Luomajoki et al. [33]	*K* = 0.51	O1 = 95.0
			O2 = 100
Sitting knee extension	Luomajoki et al. [33]	*K* = 0.95	O1/O2 = 100
Standing back extension test	Gondhalekar et al. [31]	*K* = 0.87	96
Standing knee-lift test (SKL)	Granström et al. [32]	ICC= 0.57–0.75	Hip hitch (HH): Instead of lifting the thigh up in the sagittal plane, the pelvis tilts in the frontal plane.
		*K* = 0.42–0.79 for hip hitch	
		*K* = 0.63–0.95 for lateral sway	Lateral sway (LS) of the pelvis on the stance leg.
		*K* = 0.79-0.89 for trunk lateral flexion	Trunk lateral flexion (TLF) to either side.
		*K* = 0.42–0.84 for knee not lifted straight up	Knee is not lifted straight up (KNLSU).
		*K* = 0.76–1.00 for arm lowering	One arm is lower (AL) on one side.
		*K* = 0.89–1.00 for back extension	The back extends (BE) during the movement.
		*K* = 0.61–1.00 for back flexion	The back flexes (BF) during the movement.
Static lunge test (SL)	Granström et al. [32]	ICC = 0.54-0.87	
		*K* = 0.42–0.89 for trunk lateral flexion	
		*K* = 0.95–1.00 for arm lowering	
		*K* = 0.63–0.74 for knee moving inwards	The front knee moves inwards (KMI) and not aligned with the hip and foot.
		*K* = 0.63–0.95 for pelvic tilt	The pelvis tilts (PT) to either side and not horizontally aligned.
		*K* = 0.53–0.89 for hips backwards	
			The hips move backwards (HMB) instead of downwards. The back seems to arch.
Waiter’s bow/trunk flexion	Luomajoki et al. [33]	*K* = 0.88	O1 = 97.5
			O2 = 100

NB: Tests are described in the table “Inter-examiner reliability”. Legend: O = Observation, R = Right, L = Left, Ext = Extension, Rot = Rotation.

**Table A4:** Validity of clinical tests.

Test	Authors	Validity	Notes and Summary of Results
6 tests battery:	Luomajoki et al. [39]	Effect size (ES) for the difference between the groups: 1.18 (CI 95%: 1.02–1.34).	Physiotherapists valued the performance of the subjects on the six movement control tests resulting in a score of 0–6 positive tests.
Waiter’s bow		*p* < 0.001 LBP vs healthy controls.	Authors compared the mean number of positive tests in the two groups. The differences between the groups were analyzed by the effect size (ES).
Pelvic tilt			The statistical test showed that this was a significant difference (*p* < 0.001).
		Between all the group:	
		*p* < 0.02	
		*p* < 0.01 acute vs chronic	A subgroup analysis was performed of the number of positive tests depending on LBP.
		*p* < 0.03 subacute vs chronic	A statistically significant difference was also found between acute and chronic (*p* < 0.01) as well as between subacute and chronic (*p* < 0.03). No difference between acute and subacute patient groups (*p* > 0.7).
One leg stance			
Sitting knee extension			
		*p* > 0.7 acute and subacute.	
Rocking 4 points kneeling			
Prone lying active knee flexion			
Knee lift abdominal test (KLAT)	Roussel et al. [40]	*p* = 0.048 (R/L)	The tests were performed in supine position and monitored with a pressure biofeedback unit (PBU): maximal pressure deviation from baseline was recorded during each test. The aim was to have as little deviation as possible.
Bent knee fall out (BKFO)	Roussel et al. [40]	*p* = 0.049 (L), 0.304 (R)	Significant differences were observed between dancers with and without a history of LBP (*p* value <0.05 bilaterally for KLAT and on the left leg for the BKFO).
Prone hip extension (PHE)	Bruno et al. [29]	*p* < 0.001 LBP group-patient score	The following analyses were performed:
		*p* = 0.30 patient score-examiner classification	→ exam of the effects of group status (LBP/control) and examiner classification (positive/negative) on the participant-reported perception of difficulty scores (0–5)
		*p* = 0.96 LBP group—ex classification.	→ The sensitivity (LBP group) and specificity (control group) were calculated for different cut-offs used to distinguish “positive” and “negative” participant scores.
		Sn = 0.82	
		Sp = 0.69	
		(cut-off 0–1)	
Active straight leg raise (ASLR)	Bruno et al. [29]	*p* < 0.001 LBP group-patient score	For both PHE and ASLR tests, a significant difference (*p* < 0.001) was found between the groups (LBP group perceived significant difficulty compared to the control group) but not for examiner classification. Not significant
		*p* = 0.54 patient score-examiner classification	
		*p* = 0.89 LBP group—ex classification	For both tests, the sum of sensitivity and specificity was highest with a cut-off of 0–1: Values are reported beside.
		Sn = 0.60	
		Sp = 0.76	
		(cut-off 0–1)	
Trunk forward bending and return to upright	Biely et al. [28]	For altered lumbo-pelvic rhythm (LPR):	Two different approaches for construct validity:
			(1) The ability of each individual aberrant movement to distinguish between patients with LBP, with history of LBP and without LBP.
		* *p* = 0.07	
		** *p* = 0.52	
		*** *p* = 0.23	* → LBP vs No LBP
		For deviation from sagittal plane (DEV):	** → LBP vs history of LBP
		* *p* = 0.004	
		** *p* = 0.75	*** → No LBP vs history of LBP
		*** *p* = 0.001	*p* values expressed indicate the association between the presence of aberrant movement and the presence/absence/history of low back pain.
		For instability catch (JUD):	(2) AMS:
		* *p* = 0.002	The average Aberrant Movement Score (AMS) score was calculated to provide a description
			Considering the 4 aberrant movements LPR, DEV, JUD, and painful arc of motion, the mean
		** *p* = 0.001	AMS has been calculated for each group, showing how the group that currently complains about LBP has the highest value.
		*** *p* = 0.95	
		For aberrant movement score (AMS):	The *p* values show a statistically significant difference between all groups (*p* < 0.05).
		No LBP: 0.8 ± 0.63	
		History of LBP: 1.3 ± 0.61	
		LBP: 2.5 ± 0.96	
		* *p* < 0.001	
		** *p* < 0.001	
		*** *p* = 0.021	
Standing back extension test	Gondhalekar et al. [31]	AUC: 0.785 for abdominal drawing-in maneuver (ADIM), 0.780 for ASLR	To establish validity, results of movement test from the first rater were compared with the difference in thickness during ASLR and ADIM results. Area Under the Curve (AUC) was used for assessing the validity of the standing back extension test with respect to reference standard of ultrasound measurements during ADIM and ASLR maneuvers.
			It can be between 0 and 1: the closer the curve is to the top of the graph (i.e., to 1), the greater the discriminating power of the test.
			For AUC = 0.785 and 0.780, standing back extension test can be considered moderately accurate.
Standing knee-lift test (SKL)	Granström et al. [32]	AUC: 0.47	The ability of the tests to classify the subjects into the healthy or NSLBP group was analyzed using the ROC curve quantified by using the area under the curve.
Static lunge Test (SL)	Granström et al. [32]	AUC: 0.56	Compared to the previous one, in this study, the AUC values are of lower accuracy. The authors considered an AUC of <0.5 as non-informative; 0.5 < AUC < 0.7 less accurate than chance alone; 0.7 < AUC < 0.9 moderately accurate; 0.9 < AUC < 1.0 highly accurate; and AUC = 1.0 like a perfect test.
Dynamic lunge test (DL)	Granström et al. [32]	AUC: 0.52	

Legend: Sn = Sensitivity, Sp = Specificity, ROC = Receiver Operator Characteristic. For description and criteria of tests, see table “Inter-rater reliability”.

**Table A5:** Critical appraisal tool for validity and reliability studies of objective clinical tools as described by Brink and Louw [27].

N Item	Type of Question	Nature of the study
1	If human subjects were used, did the authors give a detailed description of the sample of subjects used to perform the (index) test?	Validity and reliability studies
2	Did the authors clarify the qualification, or competence of the rater(s) who performed the (index) test?	Validity and reliability studies
3	Was the reference standard explained?	Validity studies
4	If interrater reliability was tested, were raters blinded to the findings of other rathers?	Reliability studies
5	If intrarater reliability was tested, were raters blinded to their own prior findings of the test under evaluation?	Reliability studies
6	Was the order of examination varied?	Reliability studies
7	If human subjects were used, was the time period between the reference standard and the index test short enough to be reasonably sure that the target condition did not change between the two tests?	Validity studies
8	Was the stability (or theoretical stability) of the variable being measured taken into account when determining the suitability of the time interval between repeated measures?	Reliability studies
9	Was the reference standard independent of the index test?	Validity studies
10	Was the execution of the reference standard described in sufficient detail to permit its replication?	Validity and reliability studies
11	Was the execution of the (index) test described in sufficient detail to permit replication of the test?	Validity studies
12	Were withdrawals from the study explained	Validity and reliability studies
13	Were the statistical methods appropriate for the purpose of the study?	Validity and reliability studies
